# Katanin P60: a potential biomarker for lymph node metastasis and prognosis for non-small cell lung cancer

**DOI:** 10.1186/s12957-020-01939-z

**Published:** 2020-07-06

**Authors:** Lei Wang, Jicheng Tantai, Xiaoli Zhu

**Affiliations:** 1 Department of Interventional Radiology, The First Affiliated Hospital of Soochow University, 899 Pinghai Road, Suzhou, 215006 China; 2 Department of Ultrasound, Shanghai Chest Hospital, Shanghai Jiao Tong University, Shanghai, China; 3 Department of Thoracic Surgery, Shanghai Chest Hospital, Shanghai Jiao Tong University, Shanghai, China

**Keywords:** Katanin P60, Lymph node metastasis, Survival, Tumor progression, Non-small cell lung cancer

## Abstract

**Background:**

This study aimed to assess the correlation of katanin P60 expression with clinical characteristics and survival profiles of surgical non-small cell lung cancer (NSCLC) patients.

**Methods:**

Two hundred and sixty-five primary NSCLC patients treated by surgical resection were retrospectively viewed. The expression of katanin P60 in the tumor specimen was detected by the immunohistochemical (IHC) staining assay. Preoperative clinical data were collected from patients’ medical records, and survival data were extracted from follow-up records.

**Results:**

There were 127 (47.9%) and 138 (52.1%) patients with katanin P60-low expression and -high expression, respectively; in addition, patients presenting katanin P60-high+, -high++, and -high+++ expression were 62 (23.4%), 63 (23.8%), and 13 (4.9%), respectively. Katanin P60 expression was correlated with lymph node (LYN) metastasis and advanced TNM stage but not pathological grade, tumor size, carcinoembryonic antigen (CEA) level or other non-tumor features in NSCLC patients. Regarding survival profiles, disease-free survival (DFS) and overall survival (OS) were both the lowest in katanin P60-high+++ expression patients, followed with katanin P60-high++ patients, katanin P60-high+ patients, and the highest in katanin P60-low expression patients. Further analysis illustrated that katanin P60-high expression was an independent predictive factor for unfavorable DFS and OS in NSCLC patients.

**Conclusions:**

Katanin P60 presents potential as a biomarker for lymph node metastasis and prognosis in NSCLC patients.

## Background

Lung cancer is the most commonly diagnosed malignancy as well as the leading cause of cancer-related deaths worldwide, among which non-small cell lung cancer (NSCLC) represents 85% of all lung cancer cases [1]. Although early screening has been widely applied, over a half of NSCLC patients are still diagnosed as advanced stage, leaving a limited window of curative treatments and bringing challenge to disease management [2]. Molecular biomarkers have been investigated in NSCLC to guide treatment strategy and develop treatment agents, from which, the establishment of novel therapeutic drugs have emerged [3]. The new targeted therapies, immune therapies, or alterations have shown improved survival profiles during the recent years, while the general prognosis of NSCLC is still unsatisfactory. Therefore, in order to achieve a long-term durable response and survival of NSCLC patients, it is fundamental to constantly investigate in biomarker-directed regimens and therapies.

Katanin, belonging to the family of microtubule severing enzymes, is a heterodimer with AAA catalytic subunit katanin P60 and non-AAA subunit katanin P80 [4]. Previous studies mostly focus on the role of katanin in mitosis, meiosis, and microtubule release from centrosome in the neuron, whereas only a few of them investigate the implication of katanin in malignancies [5–8]. One existing study indicates that katanin P80 correlates with larger tumor size, lymph node (LYN) metastasis, and advanced TNM stage and serves as a potential biomarker for predicting poor survival in NSCLC patients [9]. As for katanin P60, it is initially identified to be aberrantly expressed in prostate cancer patients with bone metastasis, and upregulation of katanin P60 inhibits cell proliferation but enhances cell migration [10]. In breast cancer, katanin P60 promotes cell migration, and high katanin P60 expression in tumor tissue is correlated with lymph node metastasis as well as poor overall survival (OS) in breast cancer patients [11]. As for NSCLC, only a single glimpse of study reports the regulatory role of katanin P60 in microtubule fragmentation in NSCLC cells, whereas the clinical implication of katanin P60 is poorly characterized [12]. In this study, we aimed to assess the correlation of katanin P60 with clinical characteristics and survival profiles in surgical NSCLC patients.

## Methods

### Patients

Two hundred and sixty-five primary NSCLC patients treated by surgical resection in our hospital between January 2012 and December 2014 were retrospectively analyzed in this study. The eligible subjects were (1) histopathologically confirmed as primary NSCLC, (2) TNM stage I-IIIA, (3) age ≥ 18 years, (4) underwent surgical resection, (5) tumor tissue specimens derived from resection were accessible and available for immunohistochemical (IHC) staining assay, (6) preoperative clinical features and follow-up data were complete and available, (7) no neoadjuvant therapy before surgery, (8) not relapsed NSCLC before surgery, and (9) no history of other malignancies. Approval for this study was obtained from Ethics Committee of The First Affiliated Hospital of Soochow University. The written informed consents were acquired from the patients or their family members.

### Tumor sample and clinical data collection

All tumor specimens were formalin-fixed and paraffin-embedded, which were obtained from the Pathology Department of our hospital. The expression of katanin P60 in the tumor specimen was detected by the IHC staining. In addition, preoperative clinical data including age, sex, history of smoke, history of drink, commonly chronic complications (hypertension, hyperlipidemia, diabetes, etc.), pathological grade, tumor size, LYN status, TNM stage, and carcinoembryonic antigen (CEA) level were collected from patients’ medical records. Survival data were extracted from follow-up records (to date of 2018 December 31) to evaluate the disease-free survival (DFS) and OS.

### IHC staining

Totally, 265 NSCLC samples were used for IHC staining of katanin P60. In brief, the rabbit anti-p60 katanin antibody (Abcam, USA) at 1:20 dilution and goat anti-rabbit IgG H&L (HRP) (Abcam, USA) at 1:50,000 dilution were used as primary antibody and secondary antibody, respectively, in the IHC staining. All IHC procedures were performed as described in a previous study [13]. Finally, the expression of katanin P60 in tissue specimen was assessed by IHC score, which was obtained using a semi-quantitative scoring method as previously described [14]. The total IHC score was ranging from 0 to 12. Katanin P60-high expression was defined as the IHC score > 3 in tumor slide, and katanin P60-low expression was defined as IHC score ≤ 3 in tumor slide. Additionally, katanin P60-high expression was further classified as high+ (IHC score 4–6), high++ (IHC score 7–9), and high+++ (IHC score 10–12) [15]. In addition, we detected 40 out of 265 NSCLC tissues for the expression of katanin P80. The detailed procedure of katanin P80 detection was identical to that of katanin P60, while the antibodies used were (primary antibody, rabbit anti-p80 katanin antibody at 1:1000 dilution (Abcam, UK); secondary antibody, goat anti-rabbit IgG H&L (HRP) (Abcam, USA) at 1:50,000 dilution).

### Real-time quantitative polymerase chain reaction (RT-qPCR)

For further validation, we acquired the frozen-fresh NSCLC samples (*n* = 40) that were still available in the storage to detect the katanin P60 mRNA expression using RT-qPCR. The total RNA was extracted from frozen samples by RNeasy Protect Mini Kit (Qiagen, German) following the manufacturer’s instruction and then reversely transcribed into cDNA using RT-PCR Quick Master Mix (Toyobo, Japan). Following was the fluorescent quantification using SYBR® Green Realtime PCR Master Mix (Toyobo, Japan) in ABI 7900HT Real-Time PCR System 7900 (Applied Biosystems, USA). GAPDH was used as an internal reference and katanin P60 mRNA expression was calculated by the method of 2^−ΔΔCt^. The primers used was katanin P60, forward primer (5′-3′): TGGTTCAGATGGATGGTGTTGGA; reverse primer (5′-3′): TTCTCAAGGCGTCGTCTTAAAGC; GAPDH, forward primer (5′-3′): GACCACAGTCCATGCCATCAC; reverse primer (5′-3′): ACGCCTGCTTCACCACCTT.

### Statistical analysis

Statistical analysis was carried out using SPSS 24.0 software (IBM, USA), and figure plotting was performed using GraphPad Prism 6.01 software (GraphPad Software, USA). Clinical features were described as mean and standard deviation (SD), median and interquartile (IQR), or number (percentage). Comparison of clinical features between katanin P60-high patients and katanin P60-low expression patients was determined by chi-square test or Wilcoxon rank sum test. The DFS was calculated from the date of resection to the date of disease relapse, disease progression, or death. And OS was calculated from the date of resection to the date of death. DFS and OS were analyzed using Kaplan-Meier (K-M) method. Comparison of DFS and OS between or among groups was determined by Log-rank test. Factors related to DFS and OS were analyzed by univariate and forward stepwise multivariate Cox’s proportional hazard regression model. *P* value < 0.05 was considered significant.

## Results

### Baseline characteristics

The mean age of NSCLC patients in this study was 62.0 ± 10.6 years, and the median age was 62.0 (55.0–68.0) years (Table 1). The gender composition was 205 (77.4%) males and 60 (22.6%) females. There were 150 (56.6%), 103 (38.9%), 95 (35.8%), 87 (32.8%), and 44 (16.6%) patients with a history of smoke, history of drink, hypertension, hyperlipidemia, and diabetes, respectively. As for the tumor features, the number of patients at pathological grade G1, G2, and G3 were 44 (16.6%), 160 (60.4%), and 61 (23.0%), respectively; the mean and median tumor size were 5.3 ± 2.1 cm and 5.0 (4.0–7.0) cm; 93 (35.1%) patients occurred LYN metastasis and patients at TNM stage I, II, and III were 86 (32.5%), 90 (34.0%), and 89 (33.5%), respectively. The mean and median CEA level was 47.7 ± 172.8 ng/mL and 6.2 (2.9–27.6) ng/mL, respectively.

**Table 1:** Characteristics of NSCLC patients

Items	NSCLC patients (*N* = 265)
Age (years)	
Mean ± SD	62.0 ± 10.6
Median (IQR)	62.0 (55.0–68.0)
Sex, No. (%)	
Male	205 (77.4)
Female	60 (22.6)
History of smoke, No. (%)	150 (56.6)
History of drink, No. (%)	103 (38.9)
Hypertension, No. (%)	95 (35.8)
Hyperlipidemia, No. (%)	87 (32.8)
Diabetes, No. (%)	44 (16.6)
Pathological grade, No. (%)	
G1	44 (16.6)
G2	160 (60.4)
G3	61 (23.0)
Tumor size (cm)	
Mean ± SD	5.3 ± 2.1
Median (IQR)	5.0 (4.0–7.0)
LYN metastasis, No. (%)	93 (35.1)
TNM stage, No. (%)	
I	86 (32.5)
II	90 (34.0)
III	89 (33.5)
CEA (ng/mL)	
Mean ± SD	47.7 ± 172.8
Median (IQR)	6.2 (2.9–27.6)

*NSCLC* non-small cell lung carcinoma, *SD* standard deviation, *LYN* lymph node, *CEA* carcinoembryonic antigen, *IQR* interquartile range

### Katanin P60 expression in NSCLC patients

**Fig. 1:**
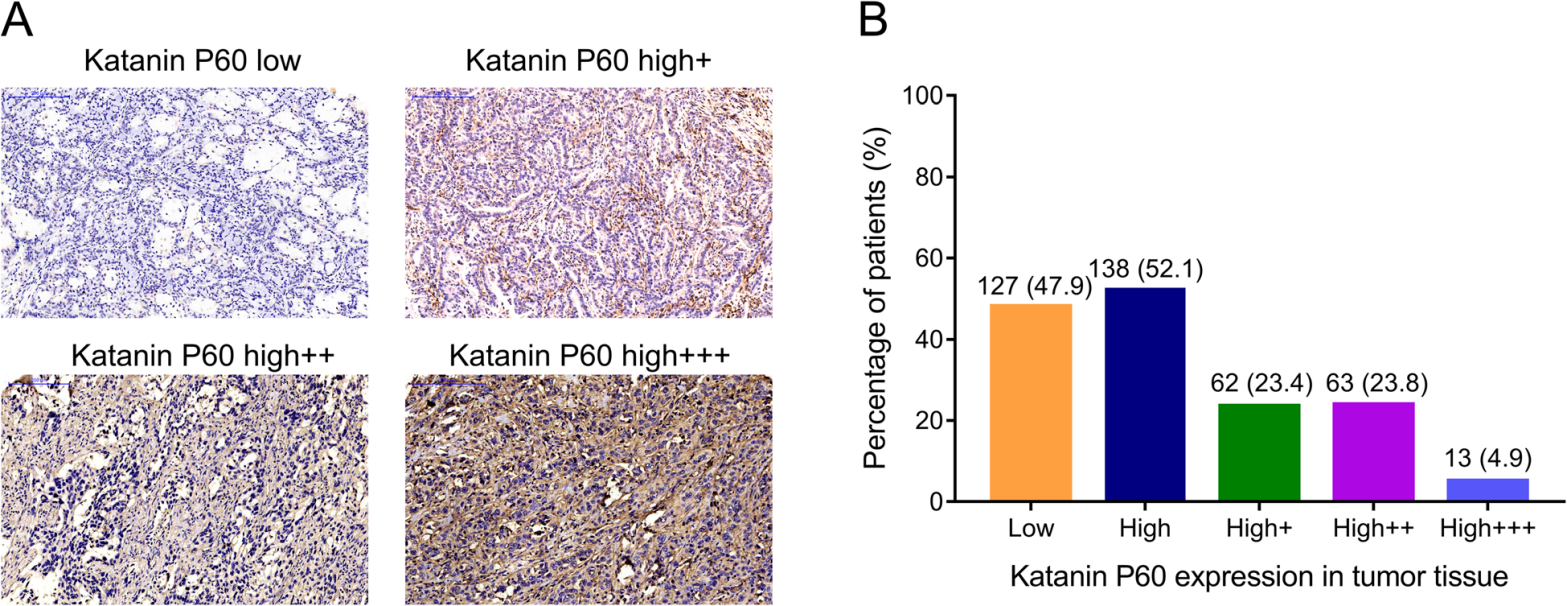
The expression of katanin P60 in NSCLC patients. IHC staining examples of NSCLC tumor katanin P60 low, high+, high++, and high+++ expression (**a**). The percentage of patients with katanin P60 low and high (further divided into high+, high++, high+++) expression (**b**). NSCLC, non-small cell lung cancer; IHC, immunohistochemistry

The expression of katanin P60 in NSCLC tissue was detected by IHC and categorized as katanin P60-low expression and katanin P60-high expression (which was further divided into high+, high++, and high+++ expression). The representative IHC images were presented in Fig. 1a. The number of patients with katanin P60-low expression and high expression were 127 (47.9%) and 138 (52.1%), respectively; in addition, patients with katanin P60-high+, -high++, and -high+++ expression were 62 (23.4%), 63 (23.8%), and 13 (4.9%), respectively (Fig. 1b).

### Correlation of katanin P60 and clinical features in NSCLC patients

**Fig. 2:**
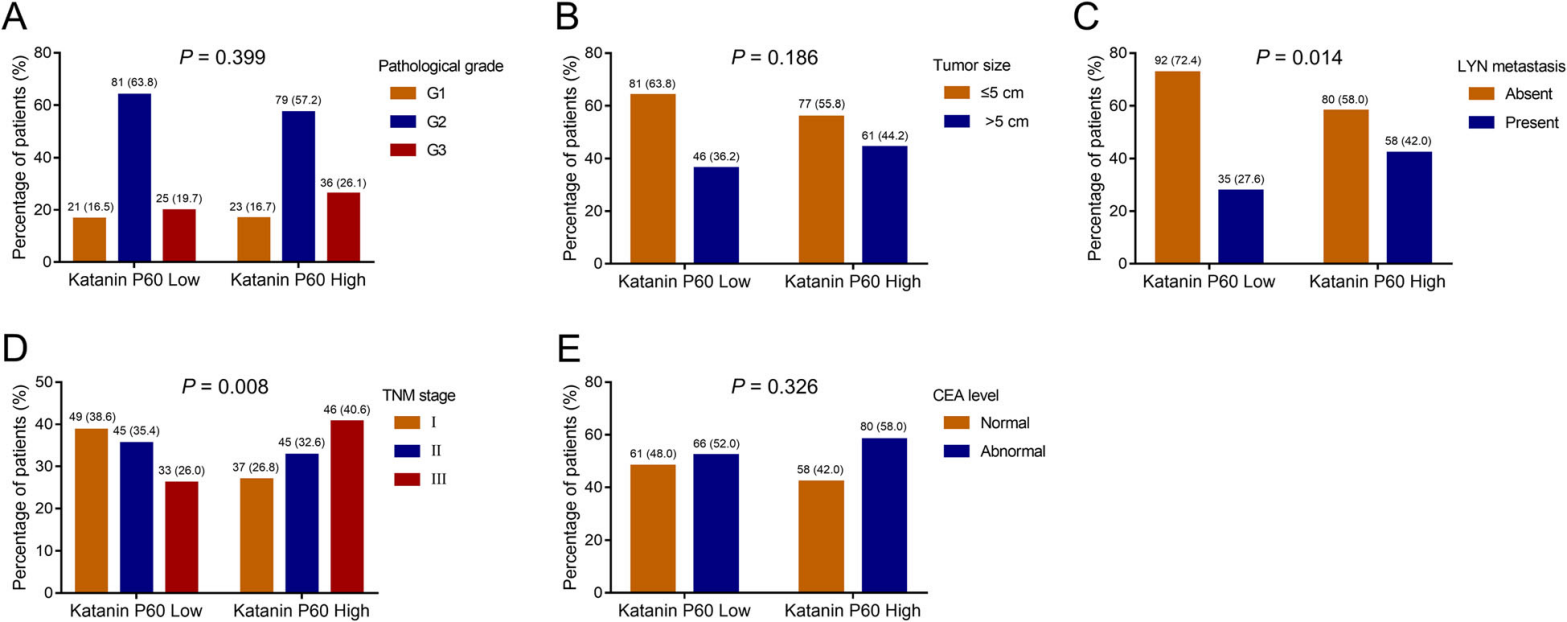
Comparison of tumor characteristics between katanin P60-high expression patients and katanin P60-low expression patients. Comparison of pathological grade (**a**), tumor size (**b**), LYN metastasis (**c**), TNM stage (**d**), CEA level between katanin P60-high expression patients, and katanin P60-low expression patients. LYN, lymph node; CEA, carcinoembryonic antigen

The number of patients with pathological grade G1, G2, and G3 were 21 (16.5%), 81 (63.8%), and 25 (19.7%) in katanin P60-low patients and 23 (16.7%), 79 (57.2%), and 36 (26.1%) in katanin P60-high patients; analysis showed no association between katanin P60 expression and pathological grade (*P* = 0.399) (Fig. 2a). Besides, 81 (63.8%) and 46 (36.2%) katanin P60-low patients had tumor size ≤ 5 cm and > 5 cm, respectively, while 77 (55.8%) and 61 (44.2%) katanin P60-high patients had tumor size ≤ 5 cm and > 5 cm, respectively; which showed that katanin P60 expression was not correlated with tumor size (*P* = 0.186) (Fig. 2b). There were 92 (72.4%) and 35 (27.6%) katanin P60-low patients with LYN metastasis absent and present, respectively while 80 (58.0%) and 58 (42.0%) katanin P60-high patients with LYN metastasis absent and present, respectively; analysis revealed that katanin P60 expression was correlated with LYN metastasis (*P* = 0.014) (Fig. 2b). The number of patients with TNM stage I, II, and III were 49 (38.6%), 45 (35.4%), and 33 (26.0%) in katanin P60-low patients and 37 (26.8%), 45 (32.6%), and 46 (40.6%) in katanin P60-high patients, which indicated that katanin P60 expression was correlated with advanced TNM stage (*P* = 0.008) (Fig. 2d). There were 61 (48.0%) and 66 (52.0%) katanin P60-low patients with normal and abnormal CEA level, respectively while 58 (42.0%) and 80 (58.0%) katanin P60-high patients with normal and abnormal CEA level respectively, which showed that katanin P60 expression was not associated with CEA level (*P* = 0.326) (Fig. 2e). In addition, katanin P60 expression was not correlated with age (*P* = 0.178), sex (*P* = 0.714), history of smoke (*P* = 0.782), history of drink (*P* = 0.176), hypertension (*P* = 0.246), hyperlipidemia (*P* = 0.856), or diabetes (*P* = 0.490) in NSCLC patients (Table 2). For further validation, katanin P60 mRNA expression was detected in 40 fresh tumor samples (Supplementary Figure 1A); then, katanin P60 mRNA high expression was also shown to be correlated with LYN metastasis (*P* = 0.038) and higher TNM stage (*P* < 0.001) but not with pathological grade, tumor size, or CEA level (all *P* > 0.05) (Supplementary Figure 1B-1F).

**Table 2:** Correlation of katanin P60 expression with demographic features and chronic complications

Items	Katanin P60 expression		*P* value
	Low (*n* = 127)	High (*n* = 138)	
Age, No. (%)			0.178
> 60.0 years	65 (51.2)	82 (59.4)	
≤ 60.0 years	62 (48.8)	56 (40.6)	
Sex, No. (%)			0.714
Male	97 (76.4)	108 (78.3)	
Female	30 (23.6)	30 (21.7)	
History of smoke, No. (%)			0.782
Yes	73 (57.5)	77 (55.8)	
No	54 (42.5)	61 (44.2)	
History of drink, No. (%)			0.176
Yes	44 (34.6)	59 (42.8)	
No	83 (65.4)	79 (57.2)	
Hypertension, No. (%)			0.246
Yes	41 (32.3)	54 (39.1)	
No	86 (67.7)	84 (60.9)	
Hyperlipidemia, No. (%)			0.856
Yes	41 (32.3)	46 (33.3)	
No	86 (67.7)	92 (66.7)	
Diabetes, No. (%)			0.490
Yes	19 (15.0)	25 (18.1)	
No	108 (85.0)	113 (81.9)	

Comparison was determined by Chi-square test

### Correlation of katanin P60 expression with DFS in NSCLC patients

**Fig. 3:**
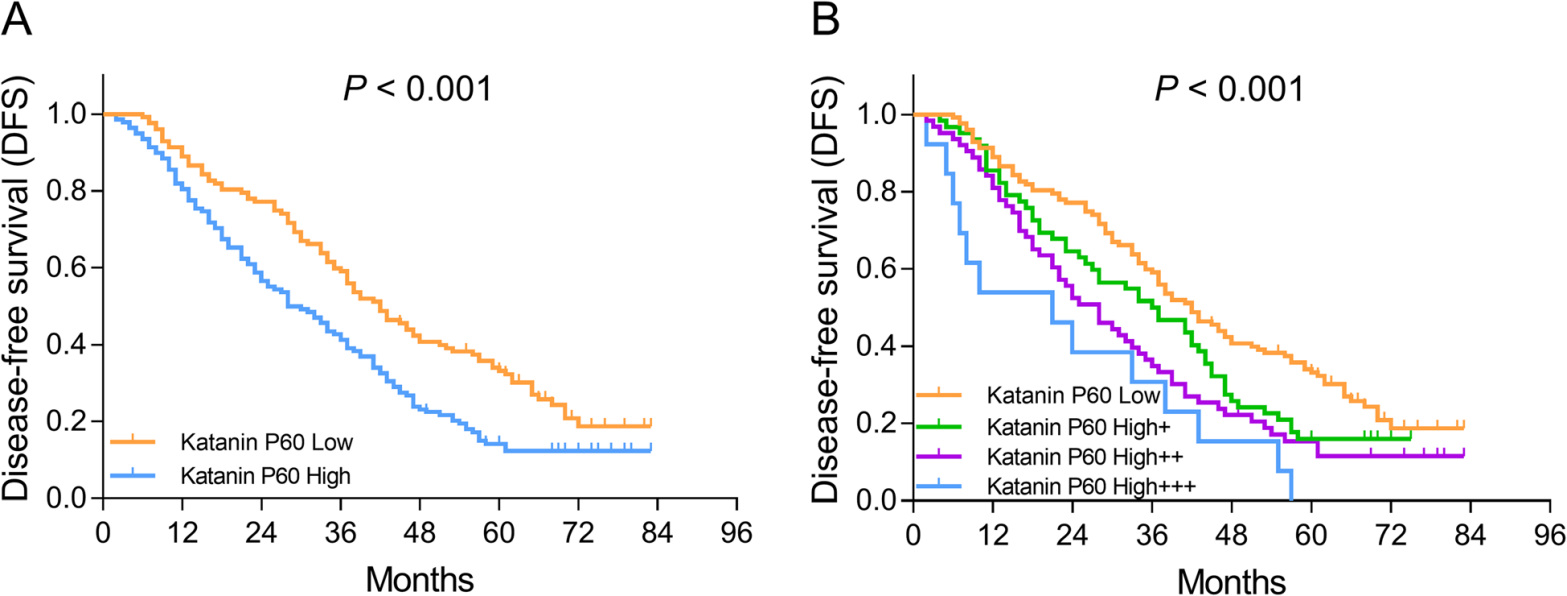
Comparison of DFS among patients with different katanin P60 expressions. Comparison of DFS between patients with katanin P60-low expression and patients with katanin P60-high expression (**a**). Comparison of DFS among patients with katanin P60-low expression, patients with katanin P60-high+ expression, patients with katanin P60-high++ expression, and patients with katanin P60-high+++ expression (**b**). DFS, disease-free survival

The DFS was shorter in katanin P60-high patients compared with katanin P60-low patients (*P* < 0.001) (Fig. 3a). In addition, patients with katanin P60 high+++ presented with the lowest DFS, followed by patients with katanin P60 high++, high+, and patients with katanin P60 low presented with the longest DFS (*P* < 0.001) (Fig. 3b).

### Factors affecting DFS in NSCLC patients

Univariate Cox’s proportional hazard regression analysis of DFS displayed that katanin P60-high expression (*P* = 0.001, HR = 1.607), tumor size (> 5.0 cm) (*P* = 0.018, HR = 1.389), LYN metastasis (*P* < 0.001, HR = 2.565), higher TNM stage (*P* < 0.001, HR = 1.524), and abnormal CEA level (*P* = 0.011, HR = 1.420) were correlated with poor DFS in NSCLC patients (Table 3). Forward stepwise multivariate Cox’s regression further disclosed that katanin P60-high expression (*P* = 0.002, HR = 1.527), LYN metastasis (*P* < 0.001, HR = 2.395), and abnormal CEA level (*P* = 0.020, HR = 1.382) were independent predictive factors for poor DFS in NSCLC patients.

**Table 3:** Cox’s proportional hazard regression analysis of DFS

Items	Cox’s regression analysis			
	*P* value	HR	95%CI	
			Lower	Higher
Univariate Cox’s regression				
Katanin P60 high	0.001	1.607	1.226	2.107
Age (> 60.0 years)	0.158	1.216	0.927	1.596
Male	0.757	1.053	0.758	1.464
History of smoke	0.844	0.973	0.744	1.274
History of drink	0.658	1.064	0.809	1.400
Hypertension	0.928	0.987	0.747	1.304
Hyperlipidemia	0.362	0.874	0.655	1.167
Diabetes	0.126	0.740	0.503	1.088
Higher pathological grade	0.061	1.218	0.991	1.498
Tumor size (> 5.0 cm)	0.018	1.389	1.059	1.823
LYN metastasis	< 0.001	2.565	1.943	3.388
Higher TNM stage	< 0.001	1.524	1.288	1.804
CEA abnormal (> 5.0 ng/mL)	0.011	1.420	1.084	1.860
Forward stepwise multivariate Cox’s regression				
Katanin P60 high	0.002	1.527	1.161	2.010
LYN metastasis	< 0.001	2.395	1.809	3.169
CEA abnormal (> 5.0 ng/mL)	0.020	1.382	1.052	1.815

*DFS* disease-free survival, *HR* hazard ratio, *CI* confidence interval, *LYN* lymph node, *CEA* carcinoembryonic antigen

### Correlation of katanin P60 expression with OS in NSCLC patients

**Fig. 4:**
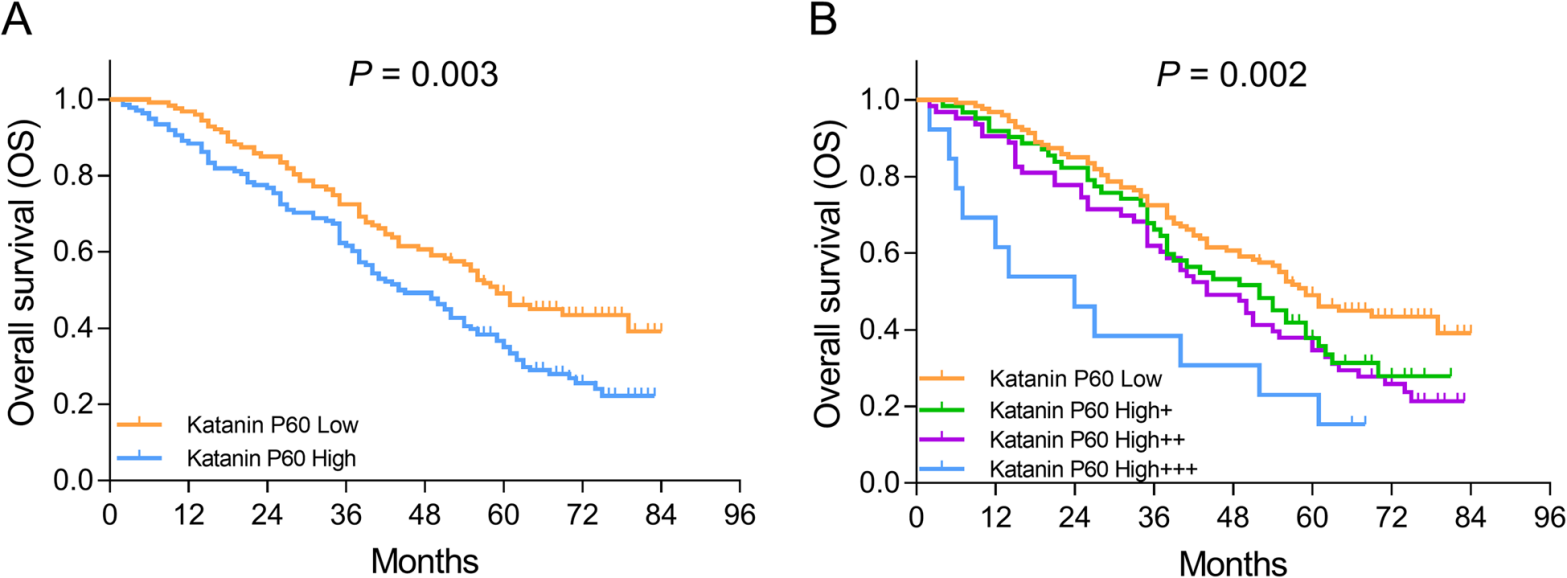
Comparison of OS among patients with different katanin P60 expressions. Comparison of OS between patients with katanin P60-low expression and patients with katanin P60-high expression (**a**). Comparison of OS among patients with katanin P60-low expression, patients with katanin P60-high+ expression, patients with katanin P60-high++ expression, and patients with katanin P60-high+++ expression (**b**). OS, overall survival

The OS was shorter in katanin P60-high patients compared with katanin P60-low patients (*P* = 0.003) (Fig. 4a). In addition, patients with katanin P60 high+++ were with the shortest OS, followed by those with katanin P60 high++, high+, and patients with katanin P60 low had the longest OS (*P* = 0.002) (Fig. 4b).

### Factors affecting OS in NSCLC patients

Univariate Cox’s proportional hazard regression analysis of OS displayed that katanin P60-high expression (*P* = 0.004, HR = 1.567), higher pathological grade (*P* = 0.001, HR = 1.508), tumor size (> 5.0 cm) (*P* = 0.008, HR = 1.499), LYN metastasis (*P* < 0.001, HR = 3.107), higher TNM stage (*P* < 0.001, HR = 1.494), and abnormal CEA (*P* = 0.001, HR = 1.668) were correlated with poor OS (Table 4). Forward stepwise multivariate Cox’s regression further disclosed that katanin P60-high expression (*P* = 0.020, HR = 1.441), higher pathological grade (*P* = 0.009, HR = 1.386), LYN metastasis (*P* < 0.001, HR = 2.801), and abnormal CEA (*P* = 0.005, HR = 1.560) were independent predictive factors for poor OS in NSCLC patients.

**Table 4:** Cox’s proportional hazard regression analysis of OS

Items	Cox’s regression analysis			
	*P* value	HR	95%CI	
			Lower	Higher
Univariate Cox’s regression				
Katanin P60 high	0.004	1.567	1.155	2.126
Age (> 60.0 years)	0.986	0.997	0.737	1.350
Male	0.469	0.877	0.616	1.250
History of smoke	0.641	1.075	0.794	1.455
History of drink	0.646	1.074	0.791	1.460
Hypertension	0.619	0.923	0.674	1.265
Hyperlipidemia	0.496	0.893	0.646	1.236
Diabetes	0.291	0.795	0.520	1.216
Higher pathological grade	0.001	1.508	1.196	1.901
Tumor size (> 5.0 cm)	0.008	1.499	1.109	2.026
LYN metastasis	< 0.001	3.107	2.287	4.221
Higher TNM stage	< 0.001	1.494	1.238	1.804
CEA abnormal (> 5.0 ng/mL)	0.001	1.668	1.225	2.270
Forward stepwise multivariate Cox’s regression				
Katanin P60 high	0.020	1.441	1.060	1.959
Higher pathological grade	0.009	1.386	1.084	1.774
LYN metastasis	< 0.001	2.801	2.057	3.814
CEA abnormal (> 5.0 ng/mL)	0.005	1.560	1.144	2.126

*OS* overall survival, *HR* hazard ratio, *CI* confidence interval, *LYN* lymph node, *CEA* carcinoembryonic antigen

## Discussion

The present study revealed that in NSCLC patients, tumor katanin P60 expression was correlated with LYN metastasis and advanced TNM stage; katanin P60-high expression was an independent predictive factor for poor DFS and OS.

Microtubule plays a vital role in regulating cell cycle, and dysregulated microtubule dynamics is correlated with abnormal cell proliferation, trafficking, signaling, and migration, which are correlated with the development and progression of malignancies [16, 17]. In lung cancer cells, inhibition of microtubule dynamics has been reported to induce G_2_/M cell cycle arrest [18]. Moreover, microtubule-regulating kinesins, such as KIF18B, are involved in accelerating cell proliferation, migration, and invasion via mediating AKT/mTOR, and thereby contribute to advanced tumor stage and correlates with unfavorable prognosis in lung adenocarcinoma patients [19]. Hence, microtubule-regulating enzymes may set off a unique and original branch for cancer research.

Katanin is an essential ATPase that degrades microtubule by severing and regulates cell movement. As one of the subunits of katanin heterodimer, katanin P60 promotes ATPase hydrolysis and depolymerizes microtubule, which contributes to tumor metastasis in several cancers. In cervical cancer, katanin P60 causes G_2_/M arrest, affects cell mitosis and tumor metastasis [20]. Katanin P60 is highly expressed in breast cancer bone metastasis tissue compared with primary tumor tissue, and overexpression of katanin P60 promotes cell migration but downregulation of katanin P60 suppresses cell migration in breast cancer cells [5]. In addition, katanin P60-high expression is reported to be correlated with LYN metastasis and poor OS in breast cancer patients [11]. In colon cancer cells, katanin P60, activated by tumor suppressor protein p53, is vital in maintaining cell survival, apoptosis, and differentiation [21]. As for prostate cancer, overexpression of katanin P60 inhibits cell proliferation but enhances cell migration, which suggests that katanin P60 accelerates prostate cancer cell metastasis [10]. The above studies illuminate the role of katanin P60 in facilitating cancer cell migration in cancers. However, limited information about the characteristics of katanin P60 in NSCLC is currently available, but one previous in vitro study mentions that katanin P60 is targeted by purine-type compound 5a that induces microtubule fragmentation and cancer cell death in lung cancer [12]. Thus, we assessed the expression and the correlation of katanin P60 with clinical features in NSCLC patients. It was observed that both katanin P60 protein and mRNA expressions were correlated with LYN metastasis and advanced TNM stage, but not pathological grade, tumor size, and CEA level in NSCLC patients. Although lacking detailed mechanisms of katanin P60 in NSCLC, the correlation of katanin P60 with LYN metastasis could be explained as follows: katanin P60 activation might stimulate cell motility and cell division via regulating microtubule dynamics and increase cell migration, thereby contributing to tumor metastasis in NSCLC [12].

Nevertheless, considering that katanin P60 is associated with poor OS in breast cancer patients, we evaluated the correlation of katanin P60 with survival profiles in NSCLC patients as well and found that katanin P60 was associated with poor DFS and OS, and further analysis illustrated that katanin P60 was an independent predictive factor for unfavorable DFS and OS in NSCLC patients [11].The possible reasons were as follows: (1) Upregulated katanin P60 might increase microtubule dynamics, accelerate cell cycle, and increase cell viability and cell migration, which promoted tumor metastasis in NSCLC and consequently led to unfavorable survival. (2) As observed in this study, katanin P60 was associated with LYN metastasis and advanced TNM stage, which were known as risk factors for poor prognosis in NSCLC patients. (3) As the cell proliferation was upregulated by katanin P60, the point mutations might be amplificated by increased number of genetic material duplication, which elevated the expression of tumor promotive genes such as *TP53*, *MYC*, and *MET*, and these genetic mutations might contribute to the development of chemoresistance and reduce the treatment response as well as survival outcomes in NSCLC patients [22]. Our findings were consistent with the previous study that katanin P80 correlates with larger tumor size, LYN metastasis, and advanced TNM stage and predicts poor survival in NSCLC patients [9]. Furthermore, there was a consistency between katanin P60 and katanin P80 expression in NSCLC, which was shown by our additional analysis (Supplementary Table 1, Supplementary Figure 2).

To our knowledge, the present study was the first that investigated katanin P60 in NSCLC progression and prognosis in clinical settings. However, despite the clinical implication, the mechanism of katanin P60 in the pathogenesis of NSCLC was not studied, which could be further explored to support our findings. Besides, only surgical NSCLC patients (TNM stage I-IIIA) were included in this study; therefore, our results might not be applicable for all stage NSCLC patients especially the advanced NSCLC patients.

## Conclusion

In conclusion, we present the initial experience that katanin P60 is sufficiently expressed and correlated with LYN metastasis and unfavorable survival profiles in NSCLC patients.

## Supplementary information

**Additional file 1:.** Figure S1. Comparison of tumor characteristics between katanin P60 high expression patients and katanin P60 low expression patients: validation by mRNA expression. MRNA expression of katanin P60 in 40 samples (A). Comparison of pathological grade (B), tumor size (C), LYN metastasis (D), TNM stage (E), CEA level (F) between katanin P60 high expression patients and katanin P60 low expression patients. LYN, lymph node; CEA, carcinoembryonic antigen.

**Additional file 2:.** Table S1. Correlation of Katanin P60 with Katanin P80 expression in NSCLC tumor t

**Additional file 3:.** Figure S2. Consistent and inconsistent expression of katanin P60 and ka0 in NSCLC tissues. NSCLC, Non-small cell lung cancer.

## Data Availability

The datasets used and/or analyzed during the current study are available from the corresponding author upon reasonable request.

## Abbreviations

NSCLC: Non-small cell lung cancer
OS: Overall survival
IHC: Immunohistochemical
LYN: Lymph node
CEA: Carcinoembryonic antigen
DFS: Disease-free survival
SD: Standard deviation
IQR: Interquartile
K-M: Kaplan-Meier
