# Supplementary material for: Katanin P60: a potential biomarker for lymph node metastasis and prognosis for non-small cell lung cancer
Source: World J Surg Oncol. 2020 Jul 6;18:157. doi: 10.1186/s12957-020-01939-z (PMC7339556; doi:10.1186/s12957-020-01939-z)
Supplement: Supplementary file 1 — Additional file 1:. Figure S1. Comparison of tumor characteristics between katanin P60 high expression patients and katanin P60 low expression patients: validation by mRNA expression. MRNA expression of katanin P60 in 40 samples (A). Comparison of pathological grade (B), tumor size (C), LYN metastasis (D), TNM stage (E), CEA level (F) between katanin P60 high expression patients and katanin P60 low expression patients. LYN, lymph node; CEA, carcinoembryonic antigen. [file 12957_2020_1939_MOESM1_ESM.pdf]

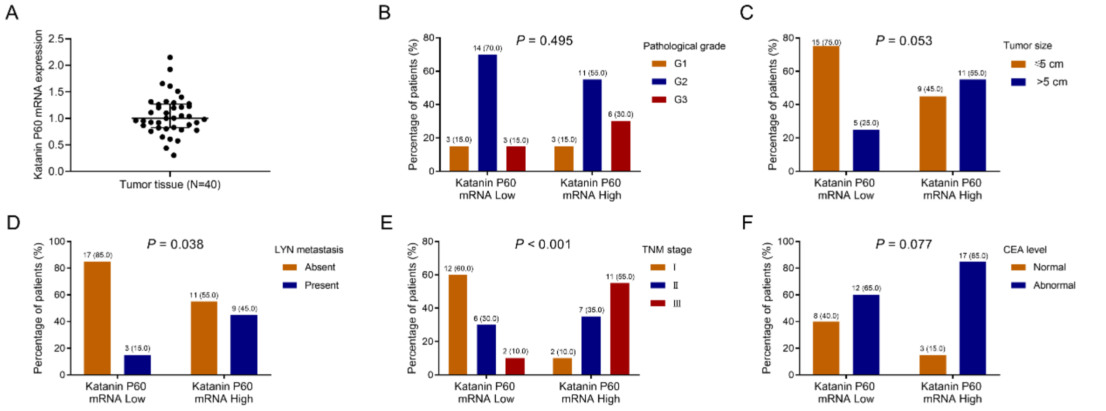

Supplementary Figure 1. Comparison of tumor characteristics between katanin P60 high expression patients and katanin P60 low expression patients: validation by mRNA expression. MRNA expression of katanin P60 in 40 samples (**A**). Comparison of pathological grade (**B**), tumor size (**C**), LYN metastasis (**D**), TNM stage (**E**), CEA level (**F**) between katanin P60 high expression patients and katanin P60 low expression patients. LYN, lymph node; CEA, carcinoembryonic antigen.
