# Supplementary material for: Katanin P60: a potential biomarker for lymph node metastasis and prognosis for non-small cell lung cancer
Source: World J Surg Oncol. 2020 Jul 6;18:157. doi: 10.1186/s12957-020-01939-z (PMC7339556; doi:10.1186/s12957-020-01939-z)
Supplement: Supplementary file 2 — Additional file 2:. Table S1. Correlation of Katanin P60 with Katanin P80 expression in NSCLC tumor t [file 12957_2020_1939_MOESM2_ESM.docx]

**Supplementary table 1.** Correlation of Katanin P60 with Katanin P80 expression in NSCLC tumor tissue

| Items |  | Katanin P60 | | Kappa coefficient | *P* value |
| --- | --- | --- | --- | --- | --- |
|  |  | Low | High |  |  |
| Katanin P80 | low | 16 (40.0) | 4 (10.0) | 0.700 | <0.001 |
|  | High | 2 (5.0) | 18 (45.0) |  |  |

NSCLC, non-small-cell lung cancer.
