# Supplementary material for: Katanin P60: a potential biomarker for lymph node metastasis and prognosis for non-small cell lung cancer
Source: World J Surg Oncol. 2020 Jul 6;18:157. doi: 10.1186/s12957-020-01939-z (PMC7339556; doi:10.1186/s12957-020-01939-z)
Supplement: Supplementary file 3 — Additional file 3:. Figure S2. Consistent and inconsistent expression of katanin P60 and ka0 in NSCLC tissues. NSCLC, Non-small cell lung cancer. [file 12957_2020_1939_MOESM3_ESM.pdf]

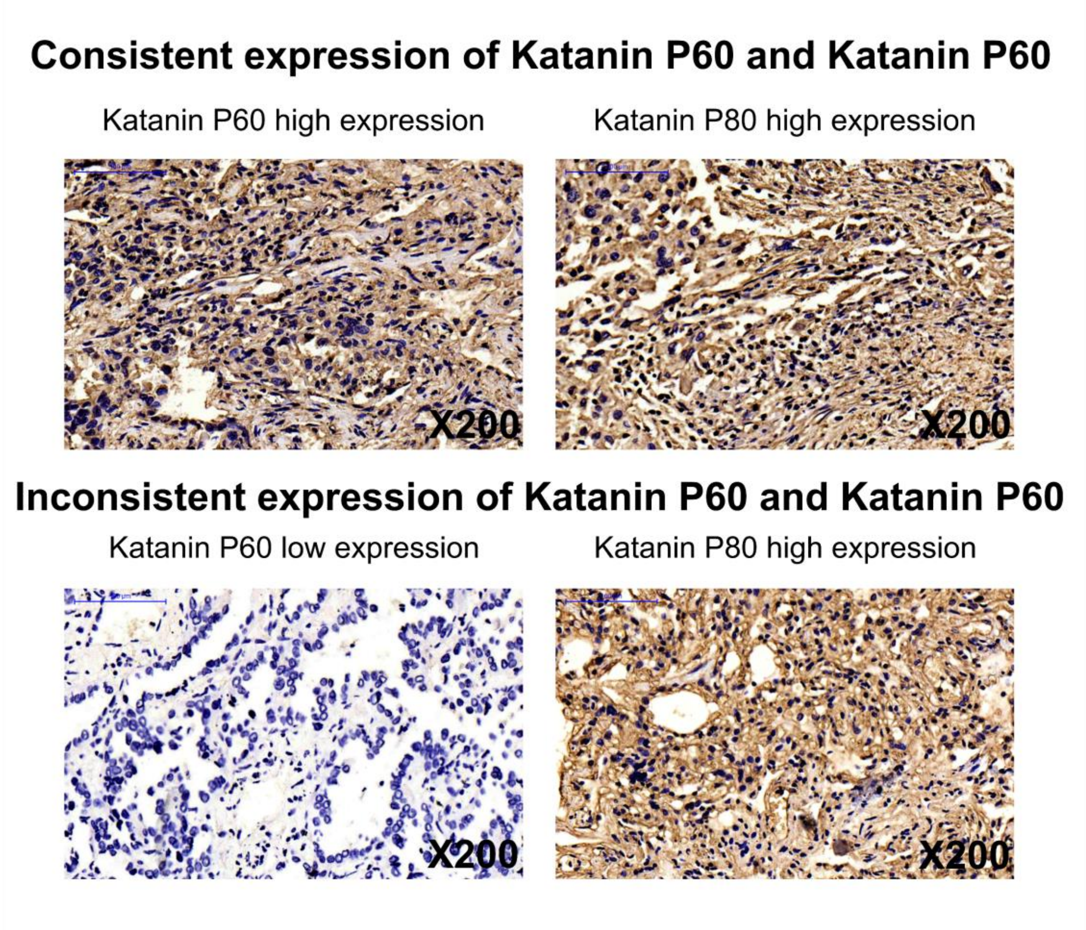

Supplementary Figure 2. Consistent and inconsistent expression of katanin P60 and katanin P80 in NSCLC tissues. NSCLC, Non-small cell lung cancer.
